# A historical budget impact and expenditure analysis of meropenem and clavulanate-based treatment for adults with XDR-TB at a specialised TB hospital in South Africa

**DOI:** 10.4102/safp.v68i1.6252

**Published:** 2026-03-04

**Authors:** Bandela B. Mgoqi, Shelley-Ann McGee, Fatima Suleman

**Affiliations:** 1 Department of Pharmaceutical Sciences, Faculty of Health Sciences, University of KwaZulu-Natal, Durban, South Africa; 2 Insight Health Solutions, Johannesburg, South Africa

**Keywords:** extensively drug-resistant tuberculosis, XDR-TB, budget impact analysis, meropenem and clavulanate, hospitalisation costs, South Africa

## Abstract

**Background:**

Extensively drug-resistant tuberculosis (XDR-TB) remains a major public health challenge in South Africa. Treatment options are costly and often require prolonged hospital stays. Assessing the cost of meropenem and clavulanate-based regimens can help guide resource allocation and policy.

**Methods:**

A retrospective chart analysis was conducted of adults aged 18–70 years with XDR-TB and treated with meropenem and clavulanate-based regimens between January 2018 and July 2023 at a specialised Tuberculosis (TB) hospital. Data sources included the hospital pharmacy dispensing system, National Health Laboratory Services billing records and the Electronic Drug-Resistant Tuberculosis Register. A budget impact model was developed from the payer’s perspective using Microsoft Excel. Descriptive and inferential statistics were applied to assess costs related to hospitalisation, medication and laboratory diagnostics.

**Results:**

A total of 62 patients met the inclusion criteria. The mean age was 39.5 years (95% confidence interval [CI]: 37.2–41.8), with 66.1% being male (*n* = 41). HIV co-infection was present in 40 patients (64.5%). The mean direct medical cost per patient for the 6-month inpatient phase was R559268.78 (≈$30 198) (95% CI: R515786.56 – R602751.00; standard deviation [s.d.]: R172653.48). Hospitalisation accounted for 87.1% of the total cost, medicines 11.9% and laboratory monitoring less than 1%, resulting in a total budget impact of R30.7 million (≈$1.66 million). Hospitalisation costs increased across the study period, and the year of initiating treatment was strongly associated with hospitalisation cost (*R*^2^ = 0.91), reflecting annual adjustments in the hospital’s patient day equivalent tariffs rather than inflation alone.

**Conclusion:**

Treatment of XDR-TB with meropenem and clavulanate-based regimens places a significant financial burden on the public health sector. Decentralised care, reduced inpatient duration and optimised procurement could reduce costs.

**Contribution:**

This study quantifies the cost of XDR-TB treatment in South Africa, highlighting hospitalisation as the main cost driver. Findings can guide improved budgeting, policy and service delivery in TB management.

## Introduction

Extensively drug-resistant tuberculosis (XDR-TB) remains a major challenge in the battle against tuberculosis (TB). Despite extensive global efforts, TB remains one of the leading causes of death worldwide, with around 10 million new cases and approximately 1.5 million deaths each year.^1^ The rise of drug-resistant strains, particularly XDR-TB, complicates the ability of healthcare professionals to effectively manage this disease and places heavy burdens on healthcare systems and economies, especially in low-resource settings.^2^

South Africa is hard hit by this issue, facing one of the highest TB burdens in the world. The country struggles significantly with drug-resistant forms of TB, including Multidrug-Resistant Tuberculosis (MDR-TB) and XDR-TB.^3^ In 2021 alone, over 177 000 TB cases were reported in South Africa, many involving resistant strains.^1^ The large number of reported TB cases highlights the urgent need for practical solutions. High rates of HIV co-infection, challenges with treatment adherence, limited healthcare resources and gaps in TB management strategies are the primary contributing factors to the rise of TB cases.^4^

Historically, treating XDR-TB has been challenging because of a lack of effective medications, severe side effects, high costs and poor patient outcomes. While newer medicines like bedaquiline and linezolid have shown promise, resistance is already emerging, emphasising the need for new therapeutic options.^5,6^ One of the promising alternatives for XDR-TB is the meropenem and clavulanate combination for patients not responding well to standard therapies.^7^

Meropenem is a carbapenem antibiotic with broad-spectrum antibacterial activity.^8^ Pairing with clavulanate, a substance that serves as a β-lactamase inhibitor to protect meropenem from degradation, enhances the effectiveness of meropenem against resistant TB strains.^9,10^ Clinical evidence from observational studies has indicated positive results with meropenem and clavulanate-based therapies, including improved culture conversion rates and better patient tolerability.^11,12^ In the South African National Department of Health (NDOH) guidelines for managing rifampicin-resistant TB, meropenem (always combined with clavulanate) is classified as a Group C agent, meaning it is reserved as an add-on drug for patients who fail or cannot tolerate other treatments.^13^ This positioning underscores both its limited eligible population and its high treatment complexity. “However, the budgetary impact of integrating meropenem and clavulanate-based regimens into routine public-sector TB care has not yet been quantified in the Eastern Cape setting.”^7^

In places with limited budgets and stretched healthcare resources, such as the Eastern Cape province, hospitals often struggle to absorb the high costs of specialised regimens like meropenem and clavulanate, requiring extended hospitalisation, skilled staff and a sustained supply of expensive medicines. Thus, understanding the implications of adopting these new treatment regimens is important to enable politicians, health administrators and policymakers to make informed decisions.

Effective economic analyses, such as budget impact analysis (BIA), are crucial for informed decision-making and become essential tools in this context. Budget impact analysis helps healthcare administrators and policymakers evaluate whether these innovative treatment options can be realistically integrated into existing budgets. Unlike cost-effectiveness analysis, which examines long-term costs and benefits, BIA provides immediate insights into short-term affordability from the payer’s perspective. This approach enables more practical decision-making regarding healthcare budgeting and resource allocation.^14,15^

This study aims to meet these needs through an in-depth historical budget impact and expenditure analysis at a specialised TB hospital in a municipality in the Eastern Cape province, South Africa. Specifically, the study sought to: (1) quantify how many patients have been treated with meropenem and clavulanate-based regimens for XDR-TB; (2) assess whether these treatments aligned with South African Standard Treatment Guidelines; (3) estimate both direct costs associated with these treatments; and (4) evaluate the overall budget implications for integrating these therapies within the healthcare budgeting framework of a high-burden area facing resource limitations.

## Research methods and design

### Study setting and population

This study is a historical budget impact and expenditure analysis using retrospective patient-level costing at a specialised drug-resistant TB hospital in a metropolitan area within the Eastern Cape province of South Africa. This facility is one of only two designated hospitals in the province for drug-resistant TB and plays a critical role in managing complicated TB cases. This facility caters to patients with susceptible TB, who require prolonged hospitalisation, as well as those with MDR-TB, pre-extensively drug-resistant TB (Pre-XDR TB) and extensively drug-resistant TB (XDR-TB).

The hospital has a total capacity of 270 inpatient beds, with 250 beds allocated for adult patients and 20 for paediatric admissions. Over the 5-year period from January 2018 to July 2023, the hospital admitted 29 880 adult patients and 129 paediatric patients. Adult inpatient numbers have steadily declined over the years, from 6775 in 2018; 6006 in 2019 and 5120 in 2020 to 3734 in 2023. Paediatric admissions, although much lower in volume, similarly trended downward: from 34 admissions in 2018 to 40 in 2019, 16 in 2020, 13 in 2021, 12 in 2022 and 14 in 2023.^16^

The hospital functions as a referral centre for both rural and urban areas and admits patients from across the province, particularly those requiring complex care, including new DR-TB agents, surgical interventions or novel salvage regimens like meropenem and clavulanate-based therapy. As per South African NDOH DR-TB guidelines,^13^ meropenem combined with clavulanate is classified as a Group C agent and was used at this facility as a salvage regimen in patients with extensively drug-resistant TB who had failed, or were non-responsive to, the standard XDR-TB regimen. Hence, eligibility for this therapy was restricted to a small subset of patients with limited treatment options.

### Sampling and data collection

The study was a retrospective chart review study. The de-identified clinical data of all 91 patients aged 18 to 70 years, treated for XDR-TB with a meropenem and clavulanate-based regimen between January 2018 and July 2023, were extracted and analysed. The 5-year period (January 2018 to July 2023) was chosen because it reflects the experience of the hospital with meropenem and clavulanate since its introduction into clinical practice in 2018. The period also corresponds to the years in which complete pharmacy, laboratory and EDRWeb data were consistently available, allowing for a comprehensive and reliable budget impact assessment.

The data were collected from patient folders, the hospital’s pharmacy dispensing system (Rx Solution), the National Health Laboratory Services (NHLS) billing system and the Electronic Drug-Resistant Tuberculosis Register (EDRWeb), a comprehensive digital case registry for patients with DR-TB in South Africa, using an approved data collection tool. EDRWeb is a reporting database that consolidates patient-level data from facility TB registers nationwide. Hence, EDRWeb has the most exhaustive compiled information about patients with DR-TB and their corresponding treatment outcomes in South Africa.^16^

### Inclusion and exclusion criteria

The inclusion criteria of the study encompassed adult patients of any gender, aged 18 to 70 years, who were started on a meropenem and clavulanate-based regimen for treating XDR-TB. Additionally, participants were required to have received in-hospital treatment for at least 6 months and should have had no concomitant disease, such as hypertension or diabetes, except HIV.

The study excluded patients under the age of 18 years, those treated with the meropenem and clavulanate regimen who did not complete the 6-month in-hospital treatment because of non-adherence, those who refused to take medication, those with adverse effects or mortality and those started on meropenem and clavulanate-based regimens after July 2023. Patients were also excluded if incomplete data were recorded on their medical charts. The total number excluded was 29.

Patients with comorbid hypertension or diabetes were excluded because these conditions require additional monitoring, investigations and medicines unrelated to XDR-TB management and would inflate the TB-specific costing. Including such patients would also introduce confounding, as some of the observed costs would reflect management of non-TB conditions rather than the meropenem and clavulanate-based regimen. Patients who did not complete the 6-month inpatient phase were excluded because their shorter and highly variable treatment periods do not allow for consistent costing, and including incomplete episodes would underestimate the average cost of a full meropenem and clavulanate-based 6-month treatment course, as standardised by the institution. The objective of the BIA was therefore to cost a uniform 6-month inpatient episode, and the 62 included patients represent all individuals who completed this full treatment period.

### Variables for extraction

The data, obtained from electronic patient medical records, Rx Solution software and EDRWeb, were transcribed onto Microsoft Office Excel® spreadsheets. Data obtained included patient’s folder number, date of birth, age, age group, gender, DR-TB type, DR-TB regimen, treatment start date, treatment stop date, reason for stopping treatment, number of months and days on treatment, final treatment outcome, HIV status, antiretroviral (ARV) regimen, ARV start date, whether the patient was on co-trimoxazole, TB culture conversion date, previous DR-TB episodes and full blood count dates. Antiretroviral regimens were extracted exactly as documented on patient charts. Some combinations may appear atypical as a result of regimen transitions, overlapping prescriptions or documentation inconsistencies in complex DR-TB cases. These regimens were retained as recorded, without adjustment.

Outcome fields such as culture conversion and final treatment outcome were inconsistently recorded and could not be validated. As this BIA focused on direct medical costs rather than clinical effectiveness, these outcomes were not analysed or reported.

### Approach to cost methodology and analysis

The approach in this study was a bottom-up, ingredient-based costing method. Unit costs were multiplied by quantities used for each patient over a standardised 6-month treatment duration. Costs were sourced directly from hospital records, state tender price lists and national health tariffs. All costs were captured in South African Rand (ZAR) and reported at actual-year values. No inflation adjustments were made as each year’s costs were analysed within their respective time frames. The exact price from the year in which the patient received treatment was used in the analysis, rather than adjusting all prices to a single base year. Microsoft Excel was used to develop the budget impact model and calculate descriptive and comparative statistics.

Length of stay (LOS) was standardised to 180 days because this reflects the routine clinical practice of the hospital for the intensive phase of meropenem and clavulanate-based therapy. Length of stay data for patients who discontinued early were highly variable and incomplete, and using these partial episodes would not allow for consistent costing. This standardisation ensures comparability across patients but does not reflect individual variation in LOS.

Medicine prices for TB regimens were sourced from the Master Procurement Catalogue and the NDOH tender price list. Where there were split contracts with different suppliers, an average unit price was calculated to reflect a more representative cost. These prices were verified by using actual hospital procurement data and depot invoices. As shown in Table 1, the DR-TB medicines included in this analysis were bedaquiline, clofazimine, delamanid, ethambutol, ethionamide, isoniazid, levofloxacin, moxifloxacin, para-aminosalicyclic acid, linezolid, terizidone and the carbapenem combination of meropenem and amoxicillin/clavulanate.

**TABLE 1:** Drug-resistant tuberculosis and antiretroviral medicines cost per tablet and/or vial.

DR-TB medicine name	Cost for treatment per year (in ZAR)					
	2018	2019	2020	2021	2022	2023
Amoxicillin/clavulanate 1000 mg tablet	R2.27	R2.71	R2.71	R2.52	R2.73	R3.10
Azithromycin 500 mg tablet	R3.35	R3.96	R3.96	R3.46	R3.66	R3.46
Bedaquiline 200 mg tablet	R0.00	R28.72	R28.72	R28.72	R28.72	R29.67
Clofazimine 100 mg capsule	R9.32	R8.42	R8.42	R9.49	R9.49	R9.49
Delamanid 50 mg tablet	R0.00	R29.37	R25.00	R31.51	R31.51	R31.51
Ethambutol 400 mg tablet	R0.70	R0.63	R0.63	R0.83	R0.83	R0.83
Ethionamide 250 mg tablet	R1.22	R1.66	R1.66	R2.46	R2.46	R2.46
Isoniazid 300 mg tablet	R0.61	R0.63	R0.63	R0.53	R0.53	R0.53
Levofloxacin 250 mg tablet	R1.72	R1.78	R1.78	R2.01	R2.01	R2.01
Levofloxacin 500 mg tablet	R3.07	R2.47	R2.47	R2.87	R2.87	R2.87
Moxifloxacin 400 mg tablet	R5.06	R5.06	R5.06	R3.56	R3.56	R3.56
Linezolid 600 mg tablet	R36.69	R49.02	R49.02	R12.40	R12.40	R8.17
Meropenem 1000 mg vial	R98.63	R98.63	R98.63	R73.60	R73.69	R63.38
Para-aminosalicylic acid 4000 mg Sachet	R35.55	R35.12	R35.12	R37.55	R37.55	R37.55
Pyrazinamide 500 mg tablet	R0.53	R0.55	R0.55	R0.55	R0.55	R0.55
Terizidone 250 mg capsule	R9.27	R7.48	R7.48	R7.88	R7.88	R7.88
**ARV Medicines**						
TDF/FTC/EFV 300 mg, 200 mg, 600 mg tablet	R4.11	R4.16	R4.16	R4.16	R4.01	R2.98
ABC and 3TC; 600 mg, 300 mg tablet	R6.01	R6.09	R5.86	R5.86	R5.86	R4.33
ABC 600 mg 300 mg tablets	R2.32	R2.25	R2.25	R2.25	R1.91	R1.86
ATV 150 mg tablet	R4.70	R5.05	R5.05	R5.05	R4.31	-
EFV 600 mg tablet	R1.59	R1.94	R1.94	R1.94	R1.71	R1.70
DTG 50 mg tablet	-	R2.24	R2.09	R2.09	R2.19	R1.06
FTC and TDF, 200 mg and 300 mg tablets	R2.35	R1.99	R1.99	R1.99	R2.44	-
3TC and AZT 300/150 mg tablet	R1.64	R1.73	R1.73	R1.73	R2.12	R1.63
3TC 150 mg tablet	R0.38	R0.68	R0.68	R0.68	R0.68	R0.63
LPV/r 200 mg; 50 mg tablet	R1.51	R2.57	R2.57	R2.57	R2.21	R2.58
NVP 200 mg tablet	R0.56	R0.76	R0.76	R0.76	R0.67	R0.74
TDF 300 mg tablet	R1.47	R1.43	R1.43	R1.43	R1.43	R1.46
AZT 300 mg tablet	R1.30	R1.63	R1.63	R1.63	R1.36	R1.56

DR-TB, drug-resistant tuberculosis; ARV, antiretroviral; ABC, Abacavir; 3TC, Lamivudine; ATV, Atazanavir; EFV, Efavirenz; DTG, Dolutegravir; FTC = Emtricitabine; TDF, Tenofovir; AZT, Zidovudine; LPV/r, Lopinavir and Ritonavir; NVP, Nevirapine; ZAR, South African Rand.

Meropenem was administered intravenously through 2 g vials twice a day, and clavulanate was supplied orally as amoxicillin clavulanate 1 g tablets twice daily. These two agents formed the backbone of the salvage regimen and were among the most expensive components. Importantly, even though meropenem and amoxicillin/clavulanate formed the common foundation, patients did not receive a standardised fixed regimen. Each patient’s regimen was tailored by clinicians, based on clinical severity, prior resistance patterns and individual tolerability. This variability was captured in the costing model, in which each patient’s actual medicine regimen was costed separately using patient-level data.

Antiretroviral medicine prices were also accessed from the Master Procurement Catalogue and the published tender list of the Department of Health, as shown in Table 1.

NHLS State Price Lists were used to determine the laboratory monitoring costs. These were applied specifically by year and sourced for 2018, 2019, the combined 2020/2021 cycle and 2023.

No official price list was available for 2022, so the average prices of 2021 and 2023 were used to estimate the laboratory costs of that year. This method ensured continuity and prevented artificial inflation or underestimation in the absence of formal tariff data. As shown in Table 2, the tests covered included full blood counts, creatinine, urea and electrolytes, liver enzymes, thyroid tests, HbA1c, sputum culture, HIV viral loads, CD4 counts and drug susceptibility testing (DST) wherever applicable.

**TABLE 2:** Laboratory costs.

Laboratory test	Cost per year (in ZAR)			
	2018/2019	2020/2021	2022	2023
Full blood count (FBC)	R387.12	R451.98	R505.11	R558.24
Liver function tests (LFTs)	R779.46	R873.72	R954.12	R1034.52
Urea and electrolytes (U&E)	R1132.80	R1284.48	R1422.36	R1560.24
TSH	R73.72	R85.06	R94.13	R103.20
HbA1c	R86.61	R106.12	R114.81	R123.50
Sputum culture (MGIT/LJ)	R820.14	R943.20	R1027.90	R1112.40
Drug susceptibility testing (DST)	R348.51	R377.70	R407.65	R437.60
CD4 count	R106.86	R123.30	R132.85	R142.40
HIV viral load ×2	R602.00	R639.26	R686.73	R734.20

ZAR, South African Rand; TSH, thyroid-stimulating hormone; HbA, hemoglobin; HIV, human immunodeficiency virus.

**TABLE 3:** Patient day equivalent and mean costs per year by component.

Year	Cost (in ZAR)					
	Mean DR-TB medicines	Mean ARV medicines	Mean lab	PDE	Mean hospitalisation	Mean total
2018	R65516.93	R732.12	R4022.17	R1624.70	R292446.00	R362717.22
2019	R68602.45	R1469.68	R4219.08	R2273.00	R409140.00	R483431.21
2020	R66199.66	R1354.62	R4503.54	R3306.40	R595152.00	R667209.82
2021	R63918.17	R570.09	R4449.07	R4341.20	R781416.00	R850353.33
2022	R70863.76	R589.88	R5047.53	R3423.20	R616176.00	R692677.17
2023	R61099.06	R264.06	R5368.00	R3830.80	R689544.00	R756275.12

PDE, patient day equivalent; DR-TB, drug-resistant tuberculosis; ARV, antiretroviral; ZAR, South African Rand.

Inpatient care was costed using patient day equivalent (PDE) values supplied by the Health Information Systems unit of the hospital. The PDE represents the average cost of delivering one inpatient day in a public-sector TB hospital and includes all major expenditure components such as staff salaries, utilities, overheads, consumables, basic equipment use and shared facility and support services. These costs are calculated annually by dividing the total expenditure of the hospital by the total number of inpatient days, meaning that capital and central services are already embedded in the PDE. Because these elements are included within the PDE, no additional hospital operational costs were added separately. As meropenem is administered intravenously twice daily through a central line, patients remain hospitalised during the intensive phase for safe administration and monitoring. A standardised LOS of 180 days was applied to reflect the 6-month intensive phase used at this facility.

Costs from all four domains, namely TB medicines, ARVs, laboratory tests and hospitalisation, were added to calculate the total direct medical cost per patient. Where applicable, patient-specific factors such as HIV status were used to refine the costing. This approach ensured that the budget impact model aligned with actual clinical practice and procurement realities. The inputs and structure of this cost analysis enabled practical comparisons across years and patient groups, helping identify the aspects of treatment that are driving expenditure.

This cost analysis was conducted from the perspective of the public-sector payer, specifically the government of the province. The analysis focused on direct medical costs incurred during the intensive 6-month inpatient treatment phase for patients diagnosed with XDR-TB and treated with a meropenem and clavulanate-based regimen. Indirect costs such as lost productivity, caregiver time or patient transport were not included, as the primary aim was to quantify the actual financial burden borne by the government health system for hospitalised patients.

All costs were calculated in ZAR. To support international interpretation, equivalent US Dollar (USD) values were additionally reported for the key cost metrics. US Dollar conversions were performed by using the 2023 average exchange rate of ZAR 18.50 = $1.00. This conversion was applied for presentation only and did not influence the costing calculations or model outputs.

### Assumptions

All patients were assumed to have received a full 6-month (180-day) inpatient treatment course without interruption. Medicine dosages were standardised across the cohort, based on national clinical guidelines, and the actual regimens were documented in patient folders.^13^ HIV-positive patients were assumed to have been on a standard first-line ARV regimen unless stated otherwise in the clinical notes. The frequency of laboratory tests was based on clinical monitoring protocols and verified by using NHLS billing records. Where contract prices varied for a single medicine in a given year, an average unit price was used to reflect typical procurement practice.

### Budget impact analysis

The budget impact was estimated from the payer’s perspective by multiplying the mean direct medical cost per patient by the number of patients who completed the standardised 6-month inpatient treatment course. This approach aligns with the methodology of BIA, which aims to estimate the expected cost of funding a defined treatment episode for a specific population. Although year-specific costs were available, averaging across the cohort allowed us to derive a single per-patient estimate that is useful for forward-looking budgeting scenarios in which future patient numbers may vary.

The formula applied was: Total Budget Impact = Mean cost per patient × Number of patients.

### Sensitivity analysis

A one-way deterministic sensitivity analysis explored how fluctuations in key cost inputs would impact the overall total cost per patient. Each major cost component, such as hospitalisation, DR-TB medicine costs, laboratory investigations and ARV therapy, was varied independently by ± 25% while holding other variables constant. This approach was selected for its practicality and relevance in real-world public-sector settings, in which small shifts in unit prices or service utilisation can influence budgets significantly. A simple LOS scenario was also included by varying the standard 180-day inpatient period by ± 25% to illustrate how shorter or longer stays may influence total cost estimates.

Probabilistic sensitivity analysis and multi-scenario modelling were not performed, as this was a historical costing analysis based on observed expenditure. These methods are recommended primarily for budget impact projections based on a forward-looking model.

### Ethical considerations

Ethics approval was obtained from the University of KwaZulu-Natal’s Biomedical Research Ethics Review Committee (BREC) on 24 June 2024. The ethical clearance number is BREC/00006682/2024. Patient confidentiality was ensured, as was data security. Gatekeeper approval was obtained from the Eastern Cape Health Research Committee and the Office of the District Manager in the metropolitan city in the Eastern Cape Province.

## Results

### Study sample characteristics

A total of 91 patients diagnosed with XDR-TB were identified during the study period from January 2018 to July 2023. Following the application of the exclusion criteria outlined in the study protocol, 29 patients were excluded because of mortality, adverse effects, non-adherence, transfer out or refusal to continue treatment before completing the required 6-month hospitalisation period. The final sample in the cost analysis comprised 62 patients who completed a full 6-month course of meropenem and clavulanate-based therapy while hospitalised at the specialised TB hospital in the Eastern Cape (see Table 4 for patient characteristics).

**TABLE 4:** Patient demographics by year, HIV status, age group, and sex (*N* = 62).

Characteristic	*n*	%
**Sex**		
Female	21	33.9
Male	41	66.1
**Age group (years)**		
18 to < 25	5	8.1
26 to < 34	15	24.2
35 to < 44	23	37.1
45 to < 54	9	14.5
55 to < 64	7	11.3
65 to < 74	2	3.2
75 to < 84	1	1.6
**HIV status**	22	35.5
Negative	40	64.5
Positive	-	-
**Year of initiation**		
2018	18	29.0
2019	18	29.0
2020	2	3.2
2021	7	11.3
2022	11	17.7
2023	6	9.7

HIV, human immunodeficiency virus.

The median age of patients in the sample was 39.5 years (range of 18–75 years old). Of the 62 patients, 41 were male (66.1%), and 21 were female (33.9%). HIV co-infection was documented in 40 patients (64.5%), all of whom received concurrent ARV therapy, typically consisting of tenofovir, emtricitabine and lopinavir with ritonavir. All patients received an individualised XDR-TB regimen constructed around a backbone of intravenous meropenem and oral amoxicillin/clavulanate. Additional agents such as bedaquiline, linezolid, clofazimine, terizidone, delamanid, ethambutol, ethionamide, isoniazid, levofloxacin, moxifloxacin, para-aminosalicylic acid and pyrazinamide were included at different doses for each patient, based on clinical judgement and resistance patterns.^13^

### Costing overview

Given the right-skewed distribution of cost data, medians and interquartile ranges are reported alongside means for a more representative summary of cost variability.

### Cost of hospitalisation

Hospitalisation accounted for the single most significant cost driver across the cohort. Each patient was hospitalised for 180 days, reflecting the intensive treatment phase at this facility.

While national policy does not mandate a 6-month inpatient stay for meropenem and clavulanate-based therapy, this standardised duration reflects local practice, particularly given the requirement for twice-daily IV administration via a central line and the need for close monitoring.

**FIGURE 1:**
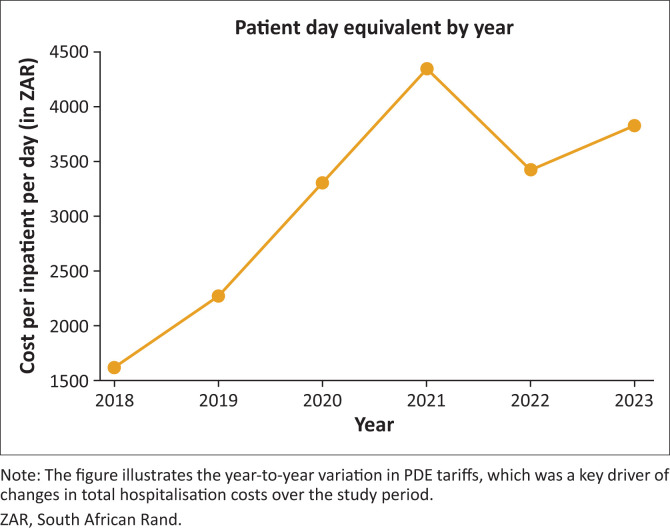
Patient day equivalent (PDE) cost per inpatient day from 2018 to 2023.

Hospitalisation costs increased sharply over the study period, driven by year-on-year changes in the patient day equivalent (PDE) tariffs of the facility (see Figure 1). The hospitalisation cost per patient rose from R292 446 in 2018 to R409 140 in 2019 (+40.0%), R595 152 in 2020 (+45.5%) and R781 416 in 2021 (+31.3%). A decline was observed in 2022 to R616 176 (−21.2%), followed by an increase to R689 544 in 2023 (+11.9%).

These fluctuations reflect changes in PDE tariffs, which are influenced by fixed overheads, operational cost pressures and shifts in inpatient volumes rather than inflation alone. Lower occupancy can increase the per-day cost of care when fixed inputs such as staffing and utilities remain constant.

### Medicine costs

The second-highest contributor to the overall cost burden was expenditure on anti-TB medicine. Medicine regimens varied widely among patients because of the salvage nature of the therapy.

Although meropenem and clavulanate were used consistently, other medicines such as bedaquiline, linezolid, terizidone and clofazimine varied among patients. Meropenem alone contributed over 50% of total medicine costs. Linezolid and bedaquiline were also significant contributors, particularly in patients with longer histories of resistance. The per-patient cost of TB medicines ranged from R38323.22 to R83682.63. This variation largely reflected differences in individual treatment regimens rather than year-specific price changes, as patients received varying combinations and durations of medicines such as linezolid, bedaquiline, terizidone and clofazimine, depending on their clinical history and resistance profile.

The mean expenditure was R66734.04, while the median was slightly higher at R69058.67, modestly skewing towards lower-cost cases. The standard deviation of R8886.53 reflects moderate variation in medicine costs among patients.

### Antiretroviral medicine costs

Antiretroviral therapy (ART) was consistently provided to all 40 HIV-positive patients throughout the 6-month TB treatment course. While the most common regimen was the combination of tenofovir, emtricitabine and lopinavir with ritonavir, the prescribed regimens varied considerably depending on prior ART history, drug–drug interaction with the DR-TB regimen and comorbidities. The most frequently used combination was tenofovir/emtricitabine with lopinavir/ritonavir, covering 16 patients at an average cost of R1840.46 per patient. TLD refers to tenofovir, lamivudine and dolutegravir, a fixed-dose antiretroviral combination, used in six patients, had the lowest average 6-month cost at R446.58. Other regimens included combinations such as Abacavir (ABC) - based treatments, Tenofovir and Emtricitabine (TDF/) with Nevirapine (NVP) or Efavirenz (EFV) and a few triple-drug regimes with Lopinavir and ritonavir (LPV/r) or Dolutegravir (DTG). This variation resulted in a broad cost range, with some patients incurring over R2000 in ARV costs. Despite this spread, ARV therapy still represented a small portion of the total treatment expenditure (see Table 5).

**TABLE 5:** Antiretroviral medicine cost per patient by regimen and HIV status.

ARV regimen	Number of patients	Cost (in ZAR)		
		Mean 6-month	Min.	Max.
ABC/3TC/LPV/r	1	R2709.24	R2709.24	R2709.24
ABC/3TC/DTG	4	R1348.32	R1335.72	R1352.52
ABC/3TC/AZT	1	R1335.66	R1335.66	R1335.66
3TC/AZT/LPV/r	1	R2304.96	R2304.96	R2304.96
TDF/3TC/ATV	1	R2114.22	R2114.22	R2114.22
TDF/3TC/DTG (TLD)	6	R446.58	R365.04	R528.12
TDF/3TC/EFV	1	R688.62	R688.62	v
TDF/EFV/LPV/r	1	R2289.90	R2289.90	R2289.90
TDF/EFV/DTG	1	R894.96	R894.96	R894.96
TDF/FTC/LPV/r	16	R1840.46	R1436.58	R2082.78
TDF/FTC/DTG	1	R806.10	R806.10	R806.10
TDF/FTC/EFV	1	R683.94	R683.94	R683.94
TDF/FTC/NVP	5	R611.53	R611.10	R613.26

ARV, antiretroviral; ABC, Abacavir; 3TC, Lamivudine; LPV/r, Lopinavir and Ritonavir; DTG, Dolutegravir; AZT, Zidovudine; TDF, Tenofovir; ATV, Atazanavir; EFV, Efavirenz; NVP, Nevirapine; TLD, Tenofovir; Lamivudine and Dolutegravir; HIV, human immunodeficiency virus; ZAR, South African Rand.

### Laboratory monitoring costs

Laboratory investigations included tests for baseline and routine monitoring, such as full blood counts, creatinine, liver function tests, CD4 count, HIV viral load and DST. The hospital does not perform routine surveillance cultures for asymptomatic colonisation with resistant bacteria, and no such tests appeared in NHLS billing records; therefore, these costs were excluded. Patients with HIV require more frequent lab investigations because of ART monitoring, adding to their lab-related expenditure. The average lab cost per patient was R4280, with HIV-positive patients showing a mean cost of R5245 compared to R3629 among HIV-negative patients (see Table 6).

**TABLE 6:** Lab cost per HIV-negative and -positive patients by year.

Laboratory test	Cost by year (in ZAR)				Mean Cost
	2018/2019	2020/2021	2022	2023	
Full blood count (FBC)	R387.12	R451.98	R505.11	R558.24	R475.61
Liver function tests (LFTs)	R779.46	R873.72	R954.12	R1034.52	R910.46
Urea and electrolytes (U&E)	R1132.80	R1284.48	R1422.36	R1560.24	R1349.97
TSH	R73.72	R85.06	R94.13	R103.20	R89.03
HbA1c	R86.61	R106.12	R114.81	R123.50	R107.76
Sputum culture (MGIT/LJ)	R820.14	R943.20	R1027.90	R1112.40	R975.91
Drug susceptibility testing (DST)	R348.51	R377.70	R407.65	R437.60	R392.87
Total lab cost (HIV −)	R3628.36	R4122.26	R4526.08	R4929.70	R4301.60
Extra monitoring test for HIV-positive patients					
CD4 count	R106.86	R123.30	R132.85	R142.40	R126.35
HIV viral load ×2	R602.00	R639.26	R686.73	R734.20	R665.55
**Total laboratory cost (HIV +)**	**R4337.22**	**R4884.82**	**R5345.66**	**R5806.30**	**R5093.50**

ZAR, South African Rand; TSH, thyroid-stimulating hormaone; HbA, hemoglobin; HIV, human immunodeficiency virus.

In practice, the laboratory monitoring schedule reflected the national *Management of Rifampicin-Resistant TB* Clinical Guidelines, which recommend baseline testing followed by monthly full blood counts, creatinine and liver function tests throughout the intensive phase of treatment. This frequency is particularly important for patients receiving linezolid- or clofazimine-containing regimens, in which haematological or hepatic toxicity may occur. For patients living with HIV, routine monitoring includes a baseline CD4 count and an HIV viral load, followed by a repeat viral load at 6 months or earlier if virological failure is suspected. Drug susceptibility testing is conducted at baseline, with repeat DST reserved for cases of clinical deterioration, non-conversion or suspected regimen failure. These guideline-driven monitoring intervals provide essential clinical context for interpreting the laboratory-related cost estimates reported in this study.

### Total mean direct medical cost

The total mean direct cost per patient for completing the 6-month inpatient phase of XDR-TB treatment was R559268.78. This amount includes all direct healthcare costs linked to admission, treatment, monitoring and discharge during that period. A breakdown of this total reveals that hospitalisation was the major cost driver, with an average of R487160.71 per patient. This factor accounted for 87.1% of the total cost, reflecting the intensive resource demands of prolonged inpatient care, including accommodation, clinical support, nursing and facility overheads, which are required to manage a condition as complex as XDR-TB.

The cost of DR-TB medicines was the next largest cost, averaging R66775.34 per patient and contributing 11.9% of the total cost. These high figures are not unexpected, as patients in this cohort were on individualised salvage regimens built around meropenem and clavulanate, often combined with other expensive agents such as linezolid and bedaquiline.

Antiretroviral medicines contributed the least, with a mean cost of R877.50 per patient (0.16% of the total). This cost corresponds to expectations, given the availability of cost-effective, fixed-dose ARV combinations on national tender. It also highlights that HIV co-infection, in terms of medicine cost, does not place a significant additional burden on inpatient budgets.

Laboratory monitoring, which is essential for guiding treatment decisions and monitoring toxicity, contributed a mean of R4455.22 or 0.8% of the total cost. While this is a smaller portion, it is a non-negotiable part of clinical management, particularly in a population that often presents with complex comorbidities. These findings reflect how public-sector resources are currently being allocated in treating XDR-TB using meropenem and clavulanate-based regimens. These findings reinforce the need for cost-conscious planning without compromising care quality, especially in provinces where XDR-TB burden remains high and hospital-based treatment is still the norm.

### Total cost per patient by component and HIV status

When costs were disaggregated by HIV status, some differences emerged. Among HIV-positive patients (*n* = 40), the mean total direct cost per patient was R539970.24, compared to R594357.04 for HIV-negative patients (*n* = 22). Although one might assume that HIV co-infection would increase treatment costs, this cohort presented slightly lower average hospitalisation costs in the HIV-positive group (R466881.75 vs. R524031.55). These differences could reflect multiple factors, including case complexity, differences in hospital LOS or timing of ART initiation.

It is worth noting, however, that the difference in group sizes may also have played a role. With only 22 patients in the HIV-negative group, individual high-cost outliers could have skewed the average upwards, particularly in categories like hospitalisation. As expected, ARV costs were observed only in the HIV-positive group, averaging R1360.13 per patient.

Laboratory costs were also higher among HIV-positive patients (R4660.90 vs. R4081.27), driven by additional monitoring such as CD4 counts and HIV viral loads. When antiretroviral therapy, laboratory monitoring and hospitalisation costs are considered together, HIV co-infection modestly increased ARV and laboratory costs but was associated with lower hospitalisation costs. This finding suggests that stable patients on antiretroviral therapy do not increase the overall financial burden of XDR-TB treatment during the inpatient phase.

### Inter-year cost variation

There was a marked year-on-year variation in the total direct medical cost of treating XDR-TB over the 6-year period. In 2018, the mean total cost per patient was R362717.22, the lowest in the cohort. Total costs increased progressively in 2019 (R483431.21) and 2020 (R667209.82), followed by a sharp rise in 2021 (R850353.33). This spike aligns with increases in the PDE tariff during that period as well as broader procurement cost pressures. In 2022, the mean total cost decreased to R692677.17 before rising again to R756275.12 in 2023. These fluctuations underscore the impact of annual tariff adjustments, supply chain variability and procurement pricing on treatment costs.

The corresponding total annual expenditure also varied substantially, reflecting both tariff changes and the number of patients treated per year. The total direct medical expenditure was R6.53 million in 2018, R8.70m in 2019, R1.33m in 2020, R5.95m in 2021, R7.62m in 2022 and R4.54m in 2023. These figures provide a clearer picture of the budgetary implications associated with annual fluctuations in patient volumes and treatment inputs.

### Budget impact

The total budget impact for treating all individuals who completed the 6-month inpatient phase was R30.7m (≈ $1.66m), using the mean per-patient cost of R559268.78 (≈ $30 198) and the cohort size of 62 patients. Hospitalisation accounted for 87.1% of this expenditure, followed by medicines at 11.9%, while laboratory monitoring and ARVs contributed less than 1%. This amount reflects the total direct medical expenditure incurred over the 2018–2023 period. Year-specific mean costs are reported in Table 3 to illustrate how total expenditure varied across the study years.

### Results from the sensitivity analysis

The sensitivity analysis indicated that hospitalisation costs had the greatest impact on the overall cost of treating XDR-TB. A 25% decrease in hospitalisation costs reduced the total cost per patient by 21.78%, while a 25% increase produced a similar rise. In contrast, TB medicine costs had a smaller effect, with changes of ± 2.98%. Variations in laboratory monitoring and ARV costs had a negligible influence, each altering the total by less than ± 0.2% (Table 7).

**TABLE 7:** Sensitivity analysis inputs.

Component	Change (%)	Adjusted total cost (in ZAR)	Change in total cost (%)
Hospitalisation	−25	R437478.60	−21.78
Hospitalisation	25	R681058.96	21.78
TB medicines	−25	R542574.94	−2.98
TB medicines	25	R575962.62	2.98
Lab tests	−25	R558154.97	−0.20
Lab tests	25	R560382.59	0.20
ARVs	−25	R559049.40	−0.04
ARVs	25	R559488.16	0.04

ARV, antiretroviral; ZAR, South African Rand; TB, tuberculoses; Lab, laboratory.

These findings reinforce that hospital-based care remains the dominant cost driver in XDR-TB treatment. Any interventions that reduce inpatient duration or shift to outpatient models, without compromising care quality, could significantly ease the financial burden on the health system.

## Discussion

This study intended to estimate the financial burden associated with treating XDR-TB using a meropenem and clavulanate-based regimen at a specialised TB hospital in the Eastern Cape. The findings showed that the mean direct medical cost per patient for a 6-month inpatient course was R559268.78. Hospitalisation made up the largest share of this total, accounting for over 87% of the cost. Tuberculosis medicines, although essential, contributed just under 12%, while laboratory testing and ARVs had a minor financial impact. HIV-positive patients showed slightly lower total costs compared to those for HIV-negative patients, which may be linked to differences in admission duration or clinical stability. The cost trends varied across the years, with a steady rise from 2018, peaking in 2021 before a slight decline.

The cohort in this study was predominantly male (66.1%). This factor mirrors national and global data showing that TB affects men disproportionately, both in prevalence and in treatment initiation.^1^ Evidence suggests that men are more likely to delay seeking care and often present with advanced disease, which may increase the likelihood of developing drug resistance and requiring salvage regimens.^17,18^ The male predominance observed here is therefore not unexpected and highlights the importance of strengthening gender-sensitive interventions in TB programmes, including earlier screening and treatment initiation among men.

The sensitivity analysis confirmed that hospitalisation remains the most sensitive cost driver.

The conclusion was based on the average per-patient cost across all study years rather than year-specific estimates. While this simplified approach does not capture the effect of annual variations in hospitalisation tariffs or medicine procurement prices, it provided a practical standardised base case for testing the robustness of results.

One of the stated objectives of this study was to assess practice against the national TB guidelines. The 2019 South African NDOH guidelines classify meropenem, when combined with clavulanate, as a Group C agent reserved for use only in individualised regimens for patients who have failed or cannot tolerate other treatments.^13^ In line with this, the specialised TB hospital restricted meropenem and clavulanate use to a small subgroup of XDR-TB patients with limited therapeutic options. Regimens were constructed around a backbone of meropenem and clavulanate but varied substantially depending on resistance patterns and patient tolerability, reflecting the salvage nature intended by the guidelines. A key difference was that patients were kept in the hospital for the full 6-month intensive phase. While the guidelines do not prescribe this, the demands of central-line access, twice-daily IV meropenem and close monitoring made prolonged hospitalisation routine. This departure from the guidelines carried major cost implications, as hospitalisation accounted for over 87% of treatment costs.

One of the main strengths of this study is its real-world nature. Unlike models relying on projections or average figures, this analysis used actual patient-level data collected over 5 years and the 6 months of treatment each year, including pricing from tender lists, NHLS billing and verified hospital PDE values. The cohort was managed at a public-sector TB referral hospital, where standardised regimens were often not used because of the complex cases. Each regimen was built around a backbone of meropenem and clavulanate, but beyond that, the composition varied widely, reflecting real prescribing patterns in salvage therapy. This approach makes the findings more relevant to similar high-burden settings across South Africa and potentially other low- and middle-income countries.

The average cost per patient seen in this study aligns closely with previous studies of XDR-TB management in hospital settings. A South African cost study by Schnippel et al. showed that inpatient care for drug-resistant TB accounted for **95%** of the total treatment cost, a pattern echoed here.^19^ The high cost of prolonged hospitalisation is also consistent with findings from Loveday et al., who compared decentralised care with hospital-based treatment and found significantly higher costs linked to the latter.^20^ Notably, the current study reports a higher overall cost per patient, likely because of including meropenem, an expensive carbapenem that is not part of the standard XDR-TB regimen and is often reserved for salvage therapy.

International comparisons also show similar trends. Gomez et al. showed that regimens containing newer agents like pretomanid and linezolid, when used in the bedaquiline, pretomanid and linezolid regimen (BPaL) combination, could be cost-saving over time despite higher upfront medicine costs.^21^ Likewise, Fekadu et al.’s cost-effectiveness analysis of bedaquiline-based regimens in South Africa found that newer agents such as bedaquiline and linezolid increased medicine costs but offered better outcomes and potentially lower long-term costs through improved treatment success.^22^ These global findings support the idea that while newer salvage regimens like the one analysed here are more expensive upfront, they may still be necessary for certain patients with advanced resistance patterns.

The findings carry strong implications for health policy and budgeting. Hospitalisation is the biggest driver of cost, and strategies aimed at reducing inpatient stay, without compromising outcomes, could make treatment more sustainable. The marked year-on-year increases in PDE values, particularly between 2018 and 2020, suggest that rising hospitalisation costs may not be explained by inflation alone. Patient day equivalents are generally calculated by dividing total hospital expenditure by the number of inpatient days, meaning that declining patient admissions inflate the per-day cost of care by eroding economies of scale. In a specialised TB hospital, in which fixed operational costs remain high despite shrinking patient numbers, this trend renders prolonged inpatient care increasingly expensive. Similar concerns were raised by Masuku et al. in their BIA of MDR-TB treatment in South Africa, which highlighted the vulnerability of provincial budgets to rising inpatient and drug-related costs.^23^

The high per-patient cost observed in this study also carries important implications when considered through the lens of opportunity cost. With a mean expenditure of over R559 000 per patient for 6 months of treatment, the financial burden of one XDR-TB admission could absorb resources equivalent to several annual nurse salaries or thousands of first-line TB treatment courses. In a province such as the Eastern Cape, where the Department of Health faces competing demands across HIV, maternal and child health and primary care, directing such a large proportion of funds to prolonged hospitalisation for a single patient may not be sustainable. These trade-offs highlight the urgent need for strategies that reduce inpatient duration or shift care to more decentralised models, thereby freeing scarce resources to serve a larger patient population without compromising outcomes.

These findings further strengthen the case for decentralised and outpatient-based models of drug-resistant TB care, which have already demonstrated better cost-efficiency in other South African and global settings.^19,20^ Such approaches may allow earlier transition to facility-based or community-level care for patients on fully oral regimens and have shown success in several provinces and in WHO-supported programmes.^24^

However, decentralisation is less feasible for meropenem and clavulanate-based regimens because twice-daily IV administration requires central-line care, aseptic technique and close clinical monitoring. Long-term carbapenem exposure is also associated with an increased risk of colonisation with carbapenem-resistant Enterobacterales, which necessitates robust infection prevention and control systems. Decentralised models are therefore more applicable to patients who have completed the meropenem phase or who are receiving fully oral regimens. Policymakers could also consider pooled procurement or local manufacturing strategies to reduce medicine costs. From a budgeting perspective, these findings provide clearer insight into the cost pressures associated with managing XDR-TB using salvage regimens.

In practical terms, several operational models could help reduce reliance on prolonged inpatient care while still protecting patient safety. Ambulatory initiation and follow-up through decentralised DR-TB units and satellite sites are already supported by South Africa’s national policy framework on decentralised DR-TB care, which promotes community-level management where clinically appropriate. These approaches are also strongly aligned with WHO guidance, which recommends ambulatory and decentralised models as the preferred mode of care for DR-TB whenever the patient is clinically stable and adequate support systems are in place.

Community-based care delivered through primary healthcare clinics, mobile DR-TB teams and community caregivers can support treatment supervision, symptom monitoring, adherence support and psychosocial follow-up. For patients eligible for fully oral regimens, digital adherence tools such as electronic medication monitors, SMS reminders and virtual check-ins can strengthen outpatient management and continuity of care. In higher-capacity settings, infusion day-wards or closely supervised outreach services could be explored for carbapenem administration although these would require robust infection prevention and control systems, central-line expertise and careful piloting. These operational strategies highlight potential avenues for reducing inpatient burden while maintaining high standards of care.

Clinicians and pharmacists must weigh the benefits of prolonged hospital care against the financial impact. While the intention is always to provide the best clinical outcome, this must be balanced with the need to use limited resources wisely. Pharmacists, in particular, have a key role in ensuring cost-effective procurement and stock management and advising on therapeutic alternatives when budgets are constrained. In a province such as the Eastern Cape, where health budgets are already stretched, allocating more than R500 000 per patient for prolonged hospitalisation could displace other essential services, making innovation in diagnostics, shorter regimens and decentralised care not only desirable but also urgent.

Limitations must be acknowledged. Firstly, the analysis focused only on direct medical costs from the payer’s perspective. Indirect costs such as patient income loss, caregiver time and transport were not captured, and future studies should incorporate these to provide a more comprehensive view of the economic burden. The study did not capture treatment outcomes such as culture conversion, cure or mortality. As a result, while the study quantifies the financial burden of meropenem and clavulanate-based therapy, the study cannot determine whether these costs translate into improved clinical outcomes.

Secondly, while the cohort size was adequate for cost modelling, it was limited to one hospital, which may affect generalisability. Thirdly, patients who did not complete the treatment regimen or discontinued early were excluded and this may introduce survivorship bias, as these individuals generally have shorter and more variable lengths of stay. As a result, the estimated average cost in this study reflects patients who completed the full 6-month intensive phase and may not represent the cost for all individuals who initiate meropenem and clavulanate-based therapy. LOS was standardised to 180 days to allow for consistent costing across the cohort, as LOS data for early discontinuations were incomplete and highly variable. To address this uncertainty, a simple LOS scenario varying ±25% was included to illustrate how shorter or longer stays might influence the total cost.

This analysis also did not include extensive scenario modelling or probabilistic sensitivity analysis, as these methods are more appropriate for projected, model-based budget impact analyses. The deterministic and LOS scenarios were selected to reflect uncertainty within a retrospective, real-world costing framework.

This study did not report culture conversion or treatment outcomes, as these data were incomplete and fell outside the scope of a BIA, which focuses on financial consequences rather than clinical effectiveness. Lastly, costs such as human resource time, facility maintenance and cross-subsidised services were not itemised separately and were instead included within the PDE values.

Although this analysis focused on costs rather than outcomes, the published literature reports treatment success rates of approximately 45% – 55% for meropenem and clavulanate-based regimens, including South African cohorts in which success has been estimated at around 50%.^24,25,26^ This rate has important implications for interpreting the financial burden of these regimens. At a success rate of roughly 50%, the implied cost per successful outcome would be approximately double the per-patient cost estimated in this study. This situation highlights the need for careful clinical selection and for pairing costly salvage regimens with strong adherence support, rapid diagnostics and timely regimen optimisation to maximise the value of care.

The study makes the following recommendations: (1) Clinicians should transition stable XDR-TB patients to community-based care earlier, whenever safe to do so. (2) Provincial health departments must budget for higher-than-average costs when salvage regimens are used, particularly those involving meropenem. (3) The national TB costing models also need to be updated to reflect real-world costs more accurately. (4) Future research should combine cost data with treatment outcomes from patients receiving meropenem and clavulanate-based regimens. Without linking costs to actual effectiveness in this setting, the value of these regimens remains uncertain.

## Conclusion

This study quantified the direct medical costs of providing a 6-month inpatient meropenem and clavulanate-based regimen for XDR-TB at a specialised TB hospital in the Eastern Cape. The findings show that hospitalisation is the dominant cost driver, accounting for more than 80% of total expenditure, largely because of the standardised 180-day inpatient stay required at this facility. The high cost per patient highlights the substantial financial pressure on provincial budgets when salvage regimens are used.

These results point to practical measures that can help reduce costs without compromising patient outcomes. Earlier transition of clinically stable patients to decentralised or community-based care could reduce LOS and ease demand on inpatient services. Strengthening procurement efficiency for high-cost medicines, monitoring PDE tariff trends and improving forecasting systems will also support more sustainable budgeting. Together, these actions can help the health system manage the financial impact of XDR-TB treatment more effectively while maintaining the quality of care.
